# Vertebrate patatin-like phospholipase domain-containing protein 4 (*PNPLA4*) genes and proteins: a gene with a role in retinol metabolism

**DOI:** 10.1007/s13205-012-0063-7

**Published:** 2012-04-18

**Authors:** Roger S. Holmes

**Affiliations:** School of Biomolecular and Physical Sciences, Griffith University, Nathan, Brisbane, QLD 4111 Australia

**Keywords:** Patatin-like phospholipase domain containing proteins, Vertebrate PNPLA4, *PNPLA4*, X-chromosome, Comparative studies

## Abstract

At least eight families of mammalian patatin-like phospholipase domain-containing proteins (PNPLA) (E.C. 3.1.1.3) catalyse the hydrolysis of triglycerides, including PNPLA4 (alternatively PLPL4 or GS2), which also acts as a retinol transacylase and participates in retinol-ester metabolism in the body. Bioinformatic methods were used to predict the amino acid sequences, secondary and tertiary structures and gene locations for *PNPLA4* genes and encoded proteins using data from several vertebrate genome projects. *PNPLA4* genes were located on the X-chromosome for the eutherian mammalian genomes examined. Opossum (marsupial), chicken, anole lizard, clawed toad, zebrafish and lancelet *PNPLA4* genes were also identified. Most vertebrate *PNPLA4* genes typically contained six coding exons whereas the lancelet *PNPLA4* gene contained five coding exons. PNPLA4 subunits were the smallest among the PNPLA-like proteins examined containing 252–255 residues, shared >64 % sequence identities and key amino acid residues and predicted motifs, including ‘patatin’ (residues 6–176); putative catalytic dyad active site residues, Ser43 and Asp163; oxy-anion ‘hole’ residues (10–15); and conserved serine residues, which may perform structural roles for this enzyme. Predicted tertiary structures for PNPLA4 ‘patatin’ were similar to those reported for potato ‘patatin’, suggesting that it is strongly conserved during evolution. Human *PNPLA4* contained a CpG49 island within the gene promoter, a miRNA-186 binding site within the mRNA 3′-noncoding region for the *PNPLA4b* isoform and exhibited wide tissue expression at a higher than average level. These and previous studies of vertebrate *PNPLA*-like gene families have suggested that *PNPLA4* is an ancient gene in evolution which has resulted from a duplication of an ancestral invertebrate *ATGL*-like gene (encoding adipose triglyceride lipase).

**Electronic supplementary material:**

The online version of this article (doi:10.1007/s13205-012-0063-7) contains supplementary material, which is available to authorized users.

## Introduction

At least eight mammalian patatin-like phospholipase domain-containing proteins (PNPLA-like) (E.C. 3.1.1.3) and genes have been reported which encode patatin-motif containing lipases (Wilson et al. 2006; Kienesberger et al. 2009; Holmes 2012). Human *PNPLA4* (also designated as *PLPL4* or *GS2*) is localized on the X-chromosome at Xp22.3, between the genes for steroid sulfatase (*STS*) and Kallman syndrome (*KAL*) (Lee et al. 1994). Other human *PNPLA*-like genes are separately localized on the human genome, including *PNPLA1* (chromosome 6) (Mungall et al. 2003), *ATGL* (also called *PNPLA2* or adipose triglyceride lipase) (chromosome 11), *PNPLA3* (*PLPL3*) and *PNPLA5* (*PLPL5*) (chromosome 22) (Schoenborn et al. 2006), *PNPLA6* (*PLPL6* or *NTE*) (neuropathy target esterase) (chromosome 19) (Lush et al. 1998; Grimwood et al. 2004), *PNPLA7* (*PLPL7*) (chromosome 9) (Humphray et al. 2009) and *PNPLA8* (*PLPL8* or *IPLA2G*) (calcium-independent phospholipase A2-gamma) (chromosome 7) (Tanaka et al. 2000; Mancuso et al. 2000; Scherer et al. 2005).

PNPLA4 catalyses the hydrolysis of triglycerides and participates in retinol-ester metabolism in the body, with a specific role reported for this enzyme in the epidermis in regulating access to retinol from retinol-ester storage depots (Kienesberger et al. 2009; Gao and Simon 2005; Gao et al. 2009). Retinol and related retinoid compounds play key roles in the body including supporting vision (Palczewski 2011), regulating epithelial cell growth and differentiation (Long et al. 2010), contributing to the growth of bone tissue (Oki et al. 2008), immune function (Pino-Lagos et al. 2010) and the activation of tumor suppressor genes (Ye et al. 2009). This retinol-ester metabolic role is in contrast to functions reported for other PNPLA-like enzymes including ATGL (or adipose triglyceride lipase) in triglyceride hydrolysis in adipocyte and non-adipocyte lipid droplets (Zimmermann et al. 2004; Haemmerle et al. 2011); PNPLA3 in contributing to hepatic fat metabolism and non-alcoholic fatty liver disease (Romeo et al. 2008); PNPLA6 (or neuropathy target esterase) which contributes to membrane lipid homeostasis and assists in maintaining axonal integrity (Zaccheo et al. 2004; Rainier et al. 2008); and PNPLA8 which serves as a calcium-independent phospholipase A2 and catalyzes the hydrolysis of membrane phospholipids (Tanaka et al. 2000; Mancuso et al. 2000).

PNPLA4 and other members of the PNPLA-like enzymes belong to the patatin family of acyl hydrolases whose proteins are characterized by a conserved amino acid sequence of Gly-X-Ser-X-Gly at their active sites, a Ser-Asp catalytic dyad (Ser43/Asp163 for human PNPLA4) (Rydel et al. 2003; Holmes 2012) instead of the Ser-His-Asp/Glu triad reported for other lipases (Cygler and Schrag 1997) and an oxy-anion ‘hole’ providing access to the active site (Rydel et al. 2003). Although three-dimensional structural analyses have not been reported for mammalian PNPLA4, the crystal structure for human PNPLA8 (also IPLA2G or cytosolic phospholipase A2) has been described (Dessen et al. 1999) showing structural similarity to potato patatin (Rydel et al. 2003).

This paper reports the predicted gene structures and amino acid sequences for *PNPLA4* genes and proteins, including primate (human [*Homo sapiens*], chimpanzee [*Pan troglodytes*], orang-utan [*Pongo abelii*], rhesus monkey [*Rhesus mulatta*], marmoset [*Callithrix jacchus*]), other eutherian mammals (rat[*Rattus norvegicus*], horse [*Equus caballus*], cow [*Bos taurus*], dog [*Canis familiaris*]), a marsupial mammal (opossum) [*Monodelphis domestica*] and other vertebrates, including chicken [*Gallus gallus*], lizard [*Anolis carolensis*], frog [*Xenopustropicalis*], zebrafish [*Danio rerio*] and lancelet [*Branchiostoma floridae*]. Predicted secondary and tertiary structures for PNPLA4 protein subunits are also described, as well as the structural relationships of these genes and enzymes with other *PNPLA*-like gene families.

## Methods

### *PNPLA4* and other *PNPLA*-like gene and protein identification

Basic Local Alignment Search Tool (BLAST) studies were undertaken using web tools from the National Center for Biotechnology Information (NCBI) (http://blast.ncbi.nlm.nih.gov/Blast.cgi) (Altschul et al. 1990). Protein BLAST analyses used the human PNPLA4 (Gao and Simon 2005) and PNPLA-like amino acid sequences deduced from reported sequences for these genes (Schoenborn et al. 2006; Dunham et al. 1999; Lush et al. 1998; Grimwood et al. 2004; Humphray et al. 2009; Tanaka et al. 2000; Mancuso et al. 2000). Non-redundant protein sequence databases for several mammalian and other vertebrate genomes were examined using the blastp algorithm, including human (*Homo sapiens*) (International Genome Sequencing Consortium 2001); chimpanzee (*Pan troglodytes*) (Chimpanzee Sequencing and Analysis Consortium 2005); orang-utan (*Pongo abelii*) (Locke et al. 2011); rhesus monkey (*Mucaca mulatta*) (Gibbs et al. 2007), marmoset (*Callithrix jacchus*) (http://genome.ucsc.edu/cgi-bin/hgGateway?db=calJac1); horse (*Equus caballus*) (Wade et al. 2009), cow (*Bos taurus*) (The Bovine Genome Sequencing and Analysis Consortium et al. 2009); mouse (*Mus musculus*) (Mouse Genome Sequencing Consortium 2002); rat (*Rattus norvegicus*) (Rat Genome Sequencing Project Consortium 2004); dog (*Canis familiaris*) (Lindblad-Toh et al. 2005); opossum (*Monodelphis domestica*) (Mikkelsen et al. 2007); chicken (*Gallus gallus*) (International Chicken Genome Sequencing Consortium 2004); lizard (*Anolis carolensis*) (Alfoldi et al. 2011); frog (*Xenopus tropicalis*) (Hellsten et al. 2010); zebrafish (*Danio rerio*) (Sprague et al. 2005); sea squirt (*Ciona intestinalis*) (Dehal et al. 2002); and lancelet (*Branchiostoma floridae*) (Putnam et al. 2008). This procedure produced multiple BLAST ‘hits’ for each of the protein databases which were individually examined and retained in FASTA format, and a record kept of the sequences for predicted encoded PNPLA-like proteins. These records were derived from annotated genomic sequences using the gene prediction method: GNOMON (http://www.ncbi.nlm.nih.gov/genome/guide/gnomon.shtml) and predicted sequences with high similarity scores generated.

BLAT analyses were subsequently undertaken for each of the predicted PNPLA4 and other PNPLA-like amino acid sequences using the UC Santa Cruz web browser (Kent et al. 2003) with the default settings to obtain the predicted locations for each of the vertebrate *PNPLA*-like genes, including predicted exon boundary locations and gene sizes (Table 1; Supplementary Table 1). Structures for human PNPLA4 isoforms were obtained using the AceView website to examine predicted gene and protein structures to interrogate this database of human mRNA sequences (Thierry-Mieg and Thierry-Mieg 2006).

**Table 1:** *PNPLA4* and other *PNPLA*-like lipase genes and proteins ^1^RefSeq: the reference amino acid sequence; ^2^predicted Ensembl amino acid sequence; and ^3^scaffold IDs are shown; GenBank IDs are derived from NCBI sources http://www.ncbi.nlm.nih.gov/genbank/; UNIPROT refers to UniprotKB/Swiss-Prot IDs for individual PNPLA4 and other PNPLA-like lipase subunits (see http://kr.expasy.org); gene size refers to base pairs of nucleotide sequences; p*I* refers to theoretical isoelectric points; the number of coding exons are listed; ‘na’ means data not available

Animal	Species	*PNPLA* gene (other name)	Chromosome coordinates	Gene size	Exon strand	Subunit (MW)	Amino acids	p*I*	GenBank ID	UNIPROT ID	¹NCBI reference ID ²NCBI predicted ID
Human	*Homo sapiens*	*PNPLA4 (PLPL4)*	X:7,866,804–7,895,475	29,493	6 –ve	27,980	253	9.0	BC020746	P41247	¹NM_001142389.1
Chimpanzee	*Pan troglodytes*	*PNPLA4 (PLPL4)*	X:7,736,089–7,762,317	26,229	6 –ve	27,964	253	9.2	na	na	²XP_001139947.1
Orangutan	*Pongo abelii*	*PNPLA4 (PLPL4)*	X:7,601,921–7,627,933	26,013	6 –ve	28,292	255	9.3	na	na	²XP_002831412.1
Gibbon	*Nomascus leucogenys*	*PNPLA4 (PLPL4)*	³GL3937281:5,083,830–5,109,334	25,505	6 –ve	27,994	253	9.2	na	na	²XP_003261040.1
Rhesus monkey	*Macaca mulatta*	*PNPLA4 (PLPL4)*	X:5,489,556–5,514,987	25,432	6 –ve	28,105	253	9.1	na	na	²NP_001180773
Marmoset	*Callithrix jacchus*	*PNPLA4 (PLPL4)*	X:5,725,477–5,753,451	27,975	6 –ve	28,194	253	9.1	na	na	²XP_002762646.1
Rat	*Rattus norvegicus*	*Pnpla4 (Plpl4)*	X:64,019,691–64,022,515	2,825	6 –ve	27,439	252	9.1	FQ216301	na	²XP_343791.1
Guinea pig	*Cavia porcellus*	*PNPLA4 (PLPL4)*	³121:3,377,779–3,414,755	36,977	6 –ve	28,052	253	9.1	na	na	²XP_003462801.1
Horse	*Equus caballus*	*PNPLA4 (PLPL4)*	X:4,394,005–4,419,747	25,743	6 –ve	28,109	253	8.9	na	F6R9V1	²XP_001488340.1
Dog	*Canis familiaris*	*PNPLA4 (PLPL4)*	X:4,847,874–4,875,987	28,114	6 –ve	28,111	253	8.7	na	E2R3S8	²XP_548849.3
Cow	*Bos taurus*	*PNPLA4 (PLPL4)*	Un.004.9:49,923–92,708	42,786	6 –ve	28,117	253	8.7	BT021623	na	²XP_590366.2
Panda	*Ailuropoda melanoleuca*	*PNPLA4 (PLPL4)*	³GL194268.1:23,365–49,508	26,144	6 +ve	28,227	253	9.1	na	na	²XP_002929669.1
Elephant	*Loxodonta africana*	*PNPLA4 (PLPL4)*	³94:3,450,626–3,485,515	34,890	6 –ve	28,113	253	8.8	na	na	²XP_003420679.1
Pig	*Sus scrofa*	*PNPLA4 (PLPL4)*	X:3,428,888–3,458,816	29,929	6 –ve	28,452	256	9.1	na	na	¹AC071250.1
Opossum	*Monodelphis domestica*	*PNPLA4 (PLPL4)*	7:35,933,455–36,004,661	71,207	6 +ve	28,263	253	9.3	na	F7F1B0	²XP_001365352.1
Chicken	*Gallus gallus*	*PNPLA4 (PLPL4)*	1:130,120,480–130,133,840	13,361	6 +ve	28,377	253	8.6	EU419877	B3TZB7	²NP_001124212.1
Lizard	*Anolis carolensis*	*PNPLA4 (PLPL4)*	3:117,647,108–117,660,289	13,182	6 –ve	27,759	253	8.2	na	na	²XP_003218860.1
Frog	*Xenopus tropicalis*	*PNPLA4 (PLPL4)*	³430:395,676–405,924	10,249	6 +ve	28,623	255	9.0	na	F6SLH7	²XP_002939012.1
Zebrafish	*Danio rerio*	*PNPLA4 (PLPL4)*	1:31,551,478–31,565,593	14,116	6 –ve	27,769	252	9.3	BC133946	B8JKG7	¹NM_001089482.1
Lancelet	*Branchiostoma floridae*	*PNPLA4 (PLPL4)*	^4^Un:621,873,136–621,877,356	4,221	5 –ve	30,817	273	7.7	na	na	²XP_002595239.1
Human	*Homo sapiens*	*PNPLA1 (PLPL1)*	6:36,238,237–36,275,490	37,254	8 +ve	57,875	532	8.4	BC103905	Q8N8W4	²NP_001139189
Rat	*Rattus norvegicus*	*Pnpla1 (Plpl1)*	20:7,139,384–7,171,885	32,502	9 +ve	63,589	589	6.3	na	na	¹NM_001191841.1
Mouse	*Mus musculus*	*Pnpla1 (Plpl1)*	17:28,995,812–29,023,893	28,082	9 +ve	65,171	592	8.6	AK132521	Q3V1D5	¹NM_001034885.3.
Opossum	*Monodelphis domestica*	*PNPLA1 (PLPL1)*	2:275,536,646–275,586,990	50,345	9 +ve	60,027	540	6.3	na	na	²XP_001378816.2
Chicken	*Gallus gallus*	*PNPLA1 (PLPL1)*	26:1,321,888–1,327,134	5,247	8 –ve	41,612	376	8.5	na	na	²XP_425818.2
Human	*Homo sapiens*	*ATGL (PNPLA2)*	11:819,719–824,859	5,141	9 +ve	55,316	504	6.7	BC011958	Q96AD5	¹NM_023376
Rat	*Rattus norvegicus*	*Atgl (Pnpla2)*	1:201,642,058–201,646,343	4,286	9 +ve	52,567	478	6.2	AC109542	P0C548	¹NM_001108509.2.
Mouse	*Mus musculus*	*Atgl (Pnpla2)*	7:148,641,186–148,645,564	4,379	9 +ve	53,657	486	6.1	BC064747	Q8BJ56	¹NR_028142
Opossum	*Monodelphis domestica*	*ATGL (PNPLA2)*	^4^Un:45,368,040–45,372,831	4,792	9 +ve	53,547	490	6.8	na	na	²XP_001380646.2
Chicken	*Gallus gallus*	*ATGL (PNPLA2)*	5:16,838,493–16,868,610	30,118	9 –ve	53,610	483	6.7	EU419874	A8WEN5	¹NM_001113291.1
Zebrafish	*Danio rerio*	*ATGL (PNPLA2)*	³3,512:30,775–45,374	14,600	10 +ve	52,253	473	6.8	BC075928	na	¹NM_001002338.1
Sea squirt	*Ciona intestinalis*	*ATGL (PNPLA2)*	³127:40,380–41,927	1,548	1 +ve	57,387	516	8.1	AK112234	na	na
Fruit fly	*Drosophila melanogaster*	*ATGL (BRUMMER)*	3L:14,770,298–14,779,178	8,881	7 –ve	57,227	507	5.9	AY051668	Q9VUH7	NM_140466.1
Human	*Homo sapiens*	*PNPLA3 (PLPL3)*	22:44,319,792–44,342,259	22,468	9 +ve	52,865	481	6.3	BC014449	Q9NST1	¹NM_025225.2
Rat	*Rattus norvegicus*	*Pnpla3 (Plpl3)*	7:122,152,145–122,171,991	19,847	9 +ve	45,908	414	6.8	EDM15609	na	na
Mouse	*Mus musculus*	*Pnpla3 (Plpl3)*	15:83,998,304–84,016,512	18,209	9 +ve	45,772	413	6.6	BC028792	Q91WW7	¹NM_054088.3
Opossum	*Monodelphis domestica*	*PNPLA3 (PLPL3)*	8:16,368,181–16,401,317	33,137	9 –ve	51,235	460	7.5	na	na	²XP_001367550.1
Chicken	*Gallus gallus*	*PNPLA3 (PLPL3)*	1:71,223,761–71,268,760	45,000	9 +ve	56,429	509	8.9	MGC86401	na	²XP_416457.2
Human	*Homo sapiens*	*PNPLA5 (PLPL5)*	22:44,276,678–44,287,760	12,299	9 –ve	47,912	429	6.3	BC031820	Q7Z6Z6	¹NM_138814
Rat	*Rattus norvegicus*	*Pnpla5 (Plpl5)*	7:122,105,840–122,115,990	10,151	9 –ve	50,408	453	8.5	na	D3ZXU1	¹NM_001130497.1
Mouse	*Mus musculus*	*Pnpla5 (Plpl5)*	15:83,943,618–83,953,543	9,926	9 –ve	48,480	432	9.0	BC109360	Q32LZ8	¹NM_029427.1
Cow	*Bos taurus*	*PNPLA5 (PLPL5)*	5:115,038,715–115,060,288	21,574	9 –ve	50,457	455	7.5	na	na	²XP_001253781.3

### Predicted structures and properties of vertebrate PNPLA4 subunits

Alignments of predicted PNPLA4 amino acid sequences were undertaken using a ClustalW method (http://www.ebi.ac.uk/Tools/msa/clustalw2/) (Chenna et al. 2003). Predicted secondary and tertiary structures for vertebrate PNPLA4 subunits were obtained using PSIPRED (McGuffin et al. 2000) and SWISS MODEL web tools, respectively (Guex and Peitsch 1997; Kopp and Schwede 2004). The reported tertiary structure for potato patatin (Rydel et al. 2003) served as the reference for the predicted PNPLA4 tertiary structures, with a modeling range of residues 6–173. Theoretical isoelectric points and molecular weights for vertebrate PNPL4 and PNPLA-like subunits were obtained using Expasy web tools (http://web.expasy.org/compute_pi/) (Gasteiger et al. 2005). Predicted trans-membrane helices for PNPLA-like sequences were obtained using CBS web tools (Center for Biological Sequence Analysis, Technical University of Denmark) (http://www.cbs.dtu.dk/services/TMHMM/) (Moller et al. 2001). Patatin-motifs were identified for PNPLA-like sequences using web tools from the National Center for Biotechnology Information (NCBI) (http://blast.ncbi.nlm.nih.gov/Blast.cgi).

### Human *PNPLA4* gene expression and predicted gene regulation sites

The human genome browser (http://genome.ucsc.edu) (Kent et al. 2003) was used to examine GNF Expression Atlas 2 data using various expression chips for the human *PNPLA4* gene (http://biogps.gnf.org) (Su et al. 2004). Predicted CpG islands and microRNA (miRNA) binding sites for human *PNPLA4* were obtained using the UC Santa Cruz Genome Browser (http://genome.ucsc.edu).

## Results and discussion

### Alignments and biochemical features of PNPLA4 amino acid sequences

**Fig. 1:**
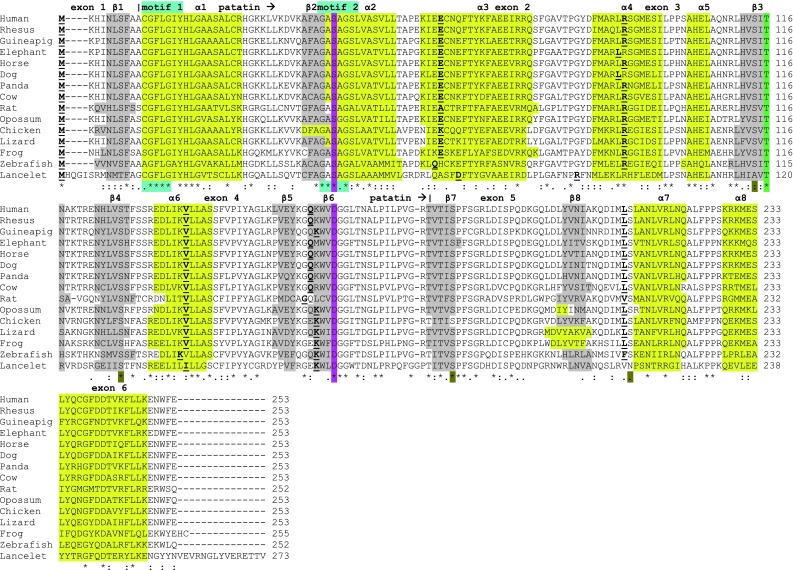
Amino acid sequence alignments for PNPLA4 sequences. See Table 1 for sources of PNPLA4 sequences; * identical residues; 1 or 2 conservative substitutions; 1 or 2 non-conservative substitutions; patatin refers to predicted motif residues (6–173); motif 1 (residues 11–15) refers to putative active site region; motif 2 refers to active site region; active site catalytic dyad residues Ser43 and Asp163; predicted helix (designated as α1, α2 etc.); predicted sheet (designated as β1, β2, etc.); conserved Thr116 and serine residues; and *bold underlined font* shows predicted exon junctions

Amino acid sequence alignments for 14 previously unreported vertebrate PNPLA4 amino acid sequences are shown in Fig. 1, together with the reported sequence for human PNPLA4 (Gao and Simon 2005; Gao et al. 2009). The PNPLA4 sequences exhibited >60 % identities, suggesting that these protein subunits are products of the same gene family, whereas the sequences for the predicted vertebrate PNPLA1, ATGL, PNPLA3 and PNPLA5 subunits were 27–37 % identical with the PNPLA4 sequences, indicating that these are members of distinct, but related *PNPLA*-like gene families (Supplementary Table 2). The sequences for the vertebrate PNPLA6, PNPLA7 and PNPLA8 subunits examined were even more distantly related with vertebrate PNPLA4 sequences with identities of <16 % (Supplementary Table 2). Two of these sequences (PNPLA6 and PNPLA7), however, showed comparatively high sequence identities (58–61 %), suggesting that these are closely related gene families. Amino acid sequences for the eight human PNPLA-like proteins examined contained 253 (PNPLA4), 429–532 (PNPLA1, PNPLA2, PNPLA3 and PNPLA5), 782 (PNPLA8) and 1,317–1,366 (PNPLA6 or NTE and PNPLA7) residues (Table 1; Supplementary Table 1). Consequently, vertebrate PNPLA4 is the smallest among these PNPLA-like proteins with an average molecular weight of ~28,000, while others exhibited MWs which are ~2 (PNPLA1, PNPLA2, PNPLA3 and PNPLA5), ~3 (PNPLA8) or ~5 times larger (PNPLA6 and PNPLA7) than PNPLA4.

Site-directed mutagenesis studies for human PNPLA4 (Gao and Simon 2005; Gao et al. 2009) and potato patatin (Hirschberg et al. 2001) have enabled the identification of key catalytic residues among those aligned for the vertebrate PNPLA4 sequences examined (Fig. 1). These included an active site motif (Gly-Xaa-Ser-Yaa-Gly designated as motif 2) (human PNPLA4 residues 41–45); active site residues Ser43 and Asp163 which serve as the catalytic dyad during catalysis; and a putative oxy-anion hole with a consensus sequence for this motif (Cys-Gly-Phe-Leu-Gly for residues 11–15 designated as motif 1). These residues are conserved among all of the vertebrate PNPLA4 sequences examined (with the exception of a Ala10 → Ser10 substitution for opossum PNPLA4), in addition to Thr116 (except for Ser116 in marmoset PNPLA4 [sequence not shown]), which is a site subject to site-specific phosphorylation (Daub et al. 2008). High theoretical isoelectric points (p*I*) were observed for each of the vertebrate PNPLA4 subunits examined (p*I* values range from 8.2–9.3), as compared with the other PNPLA-like subunits examined which exhibited lower predicted p*I* values, with the exception of the vertebrate PNPLA8 subunits (p*I* values of 9.2–9.3) (Table 1; Supplementary Table 1).

### Predicted secondary and tertiary structures for vertebrate PNPLA4 subunits

**Fig. 2:**
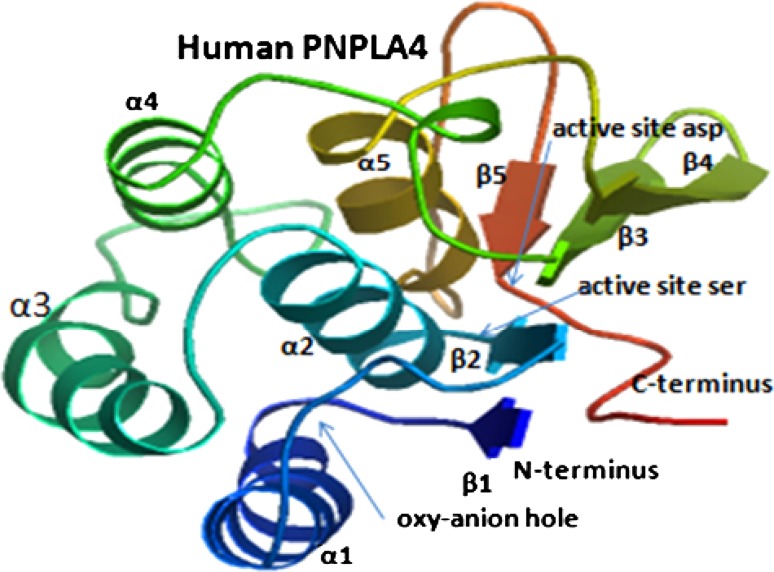
Predicted tertiary structure for human PNPLA4. The predicted structure for human PNPLA4 is based on the reported structure for potato patatin (Rydel et al. 2003) and obtained using the SWISS MODEL web site http://swissmodel.expasy.org/workspace/. The *rainbow color code* describes the 3D structures from the N- (*blue*) to C-termini (*red color*); predicted α-helices, β-sheets, active site residues (Ser43 and Asp163) and active site ‘motifs’ (1 and 2) are shown

Analyses of predicted secondary structures for PNPLA4 sequences revealed similar α-helix and β-sheet structures for all of the vertebrate subunits examined, particularly near key residues or functional domains (Fig. 1). Predicted secondary (Fig. 1) and tertiary structures (Fig. 2) were very similar to those reported for potato patatin (Rydel et al. 2003), which have been retained for all of the vertebrate PNPLA4 sequences examined. The predicted PNPLA4 tertiary structure (Fig. 2) is based on a partial sequence for this enzyme (residues 6–173) revealing the relative positioning and predicted structures for each of 5α-helices and 5β-sheets. These included the N-terminus α-helix (designated as α1), which may serve as a membrane anchor for PNPLA4 (no predicted trans-membrane properties were, however, observed for the α1 helix); an oxy-anion hole proposed for the motif previously reported (Cys-Gly-Phe-Leu-Gly for residues 11–15 designated as motif 1) located near the active site cleft (Fig. 2) which is similar to the oxy-anion hole reported for potato patatin (Rydel et al. 2003) and human PNPLA8 (encoding cytosolic phospholipase A2) (Dessen et al. 1999); a second α-helix (α2) and β-sheet (β2) which contain the active site motif Gly-Xaa-Ser-Yaa-Gly (residues 41–45 for human PNPLA4 designated as motif 2); and a β-sheet (β5) which contains Asp163, the second member of the active site dyad of catalytic residues. These structures are proximally located within a putative active site cleft supported by the predicted three-dimensional structure for this enzyme, however, any firm conclusions must await further studies. Several conserved serine residues were also observed for the vertebrate PNPLA4 sequences which may correspond to residues previously proposed for performing structural roles in potato patatin phospholipase A (Hirschberg et al. 2001; Rydel et al. 2003).

### Predicted gene locations, exonic structures and expression for vertebrate *PNPLA4* genes

Table 1 summarizes the predicted locations for vertebrate *PNPLA4* genes based on BLAT interrogations of several vertebrate genomes using the sequence for human PNPLA4 (Gao and Simon 2005; Gao et al. 2009) and the predicted sequences for other vertebrate PNPLA4 enzymes and the UC Santa Cruz Web Browser (Kent et al. 2003). Eutherian mammalian *PNPLA4* genes were located on the X-chromosome in each case, however, the marsupial *PNPLA4* gene (opossum; *Monodelphis domestica*) was located on an autosome (chromosome 7), suggesting that the X-chromosome location for *PNPLA4* is restricted to eutherian mammalian genomes. Table 1 also provides data for other vertebrate PNPLA4 genes, including the previously reported chicken PNPLA4 sequence (Saarela et al. 2008), and those predicted for lizard (*Anolis carolensis*), frog (*Xenopus tropicalis*), zebrafish (*Danio rerio*) and lancelet (*Branchiostomafloridae*) genomes, which have distinct locations to those reported here for the other vertebrate *PNPLA*-like genes. Figure 1 summarizes the predicted exonic start sites for several vertebrate *PNPLA4* genes with each having six coding exons in identical or similar positions. In contrast, lancelet *PNPLA4* contained 5 coding exons, with exon 5 corresponding to exons 5 and 6 for the vertebrate *PNPLA4* genes.

**Fig. 3:**
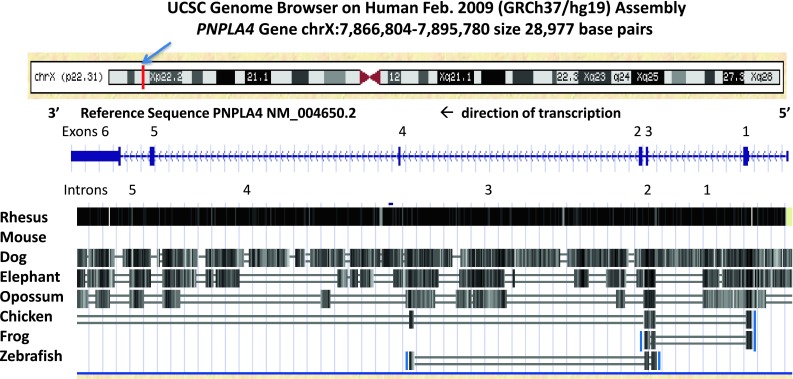
Comparative sequences for vertebrate *PNPLA4* genes derived from the UCSC Genome Browser (Kent et al. 2003) using the Comparative Genomics track to examine alignments and evolutionary conservation of *PNPLA4* gene sequences; a diagram of human chromosome X and the positioning for the human PNPLA4 gene (in *red*) was taken from the UCSC Genome Browser; genomic sequences aligned for this study included primate (human and rhesus), non-primate eutherian mammal (mouse, dog and elephant), a marsupial (opossum), bird (chicken), amphibian (frog) and fish (zebrafish); conservation measures were based on conserved sequences across all of these species in the alignments which included the 5′-untranslated, exons (exons 1–6), introns (introns 1–5) and 3′ untranslated regions for the *PNPLA4* gene; regions *shadedfromblackto grey* showing decreasing levels of sequence identity; exons 1–4 showed highest levels of gene sequence conservation

Figure 3 examined the predicted location of the human *PNPLA4* gene on the human X-chromosome as well as comparative sequence identities for vertebrate *PNPLA4* sequences. The absence of a mouse *PNPLA4* gene was readily apparent from this study. Moreover, a major decrease in sequence identities for vertebrate *PNPLA4* genes with the human *PNPLA4* gene was observed for the more distantly related species examined, especially for the intronic sequences and for exons 5 and 6 of chicken, frog and zebrafish *PNPLA4* genes. It is suggested that this may reflect a higher level of conservation for the ‘patatin’ encoding regions for the vertebrate PNPLA4 sequences, which are encoded by exons 1–4 of the vertebrate *PNPLA4* genes examined (Fig. 1).

Supplementary Table 3 examined the comparative sizes for several vertebrate PNPLA4 genes and intronic sequences (introns 1–5 for vertebrate *PNPLA4* genes and introns 1–4 for the lancelet *PNPLA4* gene examined). The rat *PNPLA4* gene was much smaller than other *PNPLA4* genes examined, being >10 times smaller than the human gene, which is reflected in the smaller sizes observed for introns 1, 3, 4 and 5. Moreover, a mouse *PNPLA4* gene was not detected in this and previous studies and further investigations are required to demonstrate whether this gene is absent from the mouse genome or has escaped detection at this stage. The guinea pig (*Cavia porcellus*) *PNPLA4* gene, however, resembled other mammalian *PNPLA4* genes in the comparative sizes of introns, which suggested that the small size for the rat *PNPLA4* gene was not a common feature for other rodent *PNPLA4* genes. Comparisons of intron sizes for vertebrate and invertebrate PNPLA4 genes also showed that intron 2 was much smaller for all mammalian (also chicken and lizard) *PNPLA4* genes examined than other introns, although intron 2 sequences for frog (*Xenopus tropicalis*), zebrafish (*Danio rerio*) and lancelet (*Branchiostoma floridae*) *PNPLA4* genes were much larger than for the mammalian *PNPLA4* genes.

**Fig. 4:**
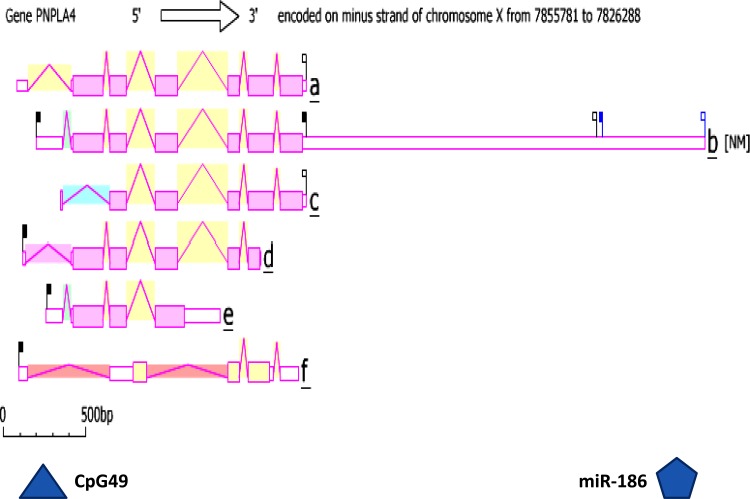
Gene structure and major isoforms for human *PNPLA4.* From AceView website (Thierry-Mieg and Thierry-Mieg 2006) http://www.ncbi.nlm.nih.gov/IEB/Research/Acembly/ mature isoform variants (designated as ‘*a*’, ‘*b*’ etc.) are shown for each *PNPLA4* transcript; capped 5′- and 3′- ends for the predicted mRNA sequences are identified; a predicted CpG49 island, a miRNA binding site (miR-186) and a scale of base pairs of nucleotide sequences are shown

**Fig. 5:**
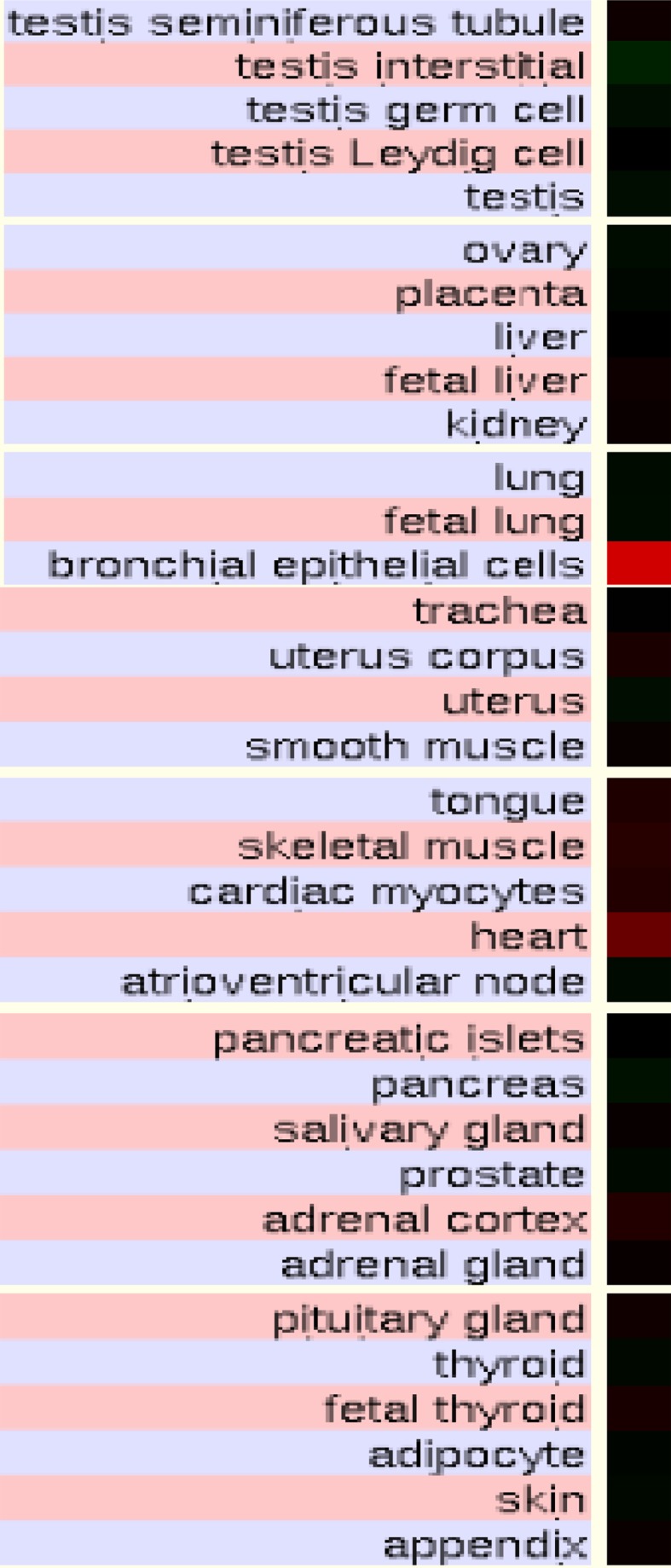
Human tissue gene expression ‘heat maps’ for *PNPLA4* taken from the human genome browser (Kent et al. 2003) (http://genome.ucsc.edu); GNF Expression Atlas 2 data using expression chips for human PNPLA4 (http://biogps.gnf.org) (Su et al. 2004); comparative gene expression levels among human tissues: *red* (high) and *black* (intermediate) expression levels

Figure 4 illustrates the comparative predicted structures of pre-messenger RNA human PNPLA4 gene transcripts (http://www.ncbi.nlm.nih.gov/IEB/Research/Acembly/) (Thierry-Mieg and Thierry-Mieg 2006). There were 6 introns present for the pre-messenger mRNA *PNPLA4a* and *PNPLA4b* transcripts, with the latter containing a CpG49 island in the 5′-noncoding segment corresponding to the promoter for this gene. In addition, the *PNPLA4b* transcript contained an extended 3′-noncoding segment with a predicted miRNA-186 binding site. These predicted gene regulation sites may contribute to the high level of gene expression (×1.5 times the expression of the average gene) and wide tissue expression observed for *PNPLA4*. Elango and Yi (2011) have previously reported that larger CpG islands are associated with gene promoters of housekeeping genes showing a broad range of gene expression and containing more RNA polymerase II binding sites than other promoters. Moreover, miRNAs are post-transcriptional regulators that bind to complementary sequences on target messenger RNA transcripts (mRNAs), usually resulting in translational repression or target degradation and gene silencing (Bartel 2009). Consequently, the presence of CpG49 and miRNA-186 within the *PNPLA4* gene may contribute significantly to the broad tissue expression observed for *PNPLA4* transcripts. Figure 5 presents ‘heat maps’ showing the comparative gene expression for various human tissues obtained from GNF Expression Atlas Data using U133A and GNF1H *PNPLA4* chips (Su et al. 2004) with higher levels being observed in bronchial epithelial cells and heart as well as significant expression in the other tissues examined.

### Phylogeny of vertebrate PNPLA4 and other PNPLA-like lipases

A phylogenetic tree has been previously described from alignments of vertebrate ATGL-like amino acid sequences (PNPLA1, ATGL, PNPLA3, PNPLA4 and PNPLA5) with the predicted fruit fly (*Drosophila melanogaster*) ATGL sequence serving to ‘root’ the tree (Holmes 2012). Clustering was reported for five major groups of vertebrate ATGL-like sequences: PNPLA1; ATGL (or PNPLA2); PNPLA3; PNPLA4; and PNPLA5. Clustering into sub-groupings was also described, including PNPLA3 and PNPLA5, with ATGL; and PNPLA4 with PNPLA1. These results were consistent with the presence of *ATGL*-like and *PNPLA4*-like genes within primitive vertebrate genomes examined, and were suggestive of an initial gene duplication event for *ATGL* generating both of these genes, during the evolutionary appearance of vertebrates. This is consistent with *PNPLA4* being an ancient gene, appearing in some primitive vertebrate genomes and being present throughout vertebrate evolution over a period of evolution of >500 million years, which is reported for the timing of the appearance of vertebrates during evolution (Donoghue and Benton 2007).

These phylogenetic studies were also extended to include other PNPLA-like genes and proteins, namely *PNPLA6*, *PNPLA7* and *PNPLA8* sequences (Holmes 2012). The results were indicative of at least three major PNPLA-like sequence groups, including the *ATGL*-like sequences (*PNPLA1*, *ATGL (PNPLA2)*, *PNPLA3*, *PNPLA4* and *PNPLA5* (Group 1); the *PNPLA6* and *PNPLA7* sequences (Group 2); and the *PNPLA8* sequences (Group 3). Group 1 sequences were further divided according to the designation of *ATGL*-like gene families, which clustered with the sea squirt *ATGL*-like sequence, and were suggestive of an ancestral relationship between early vertebrate *ATGL* and *PNPLA4* genes, with other members of *PNPLA*-like group 1 sequences, which appeared later during vertebrate evolution: *PNPLA1* and *PNPLA3/PNPLA5*. This report (Holmes 2012) also suggested that vertebrate *PNPLA6* and *PNPLA7* sequences shared a common evolutionary origin distinct to the *ATGL*-like and *PNPLA8* sequences, which were ‘rooted’ with the sea squirt (*Ciona intestinalis*) *PNPLA7* sequence, whereas the vertebrate *PNPLA8* sequences were also distinct and separately ‘rooted’ with the sea squirt (*Ciona intestinalis*) *PNPLA8* sequence.

Overall, it is likely that the three *PNPLA*-like groups of genes and proteins have arisen from distinct ancestral genes during vertebrate evolution, namely *ATGL* (the proposed ancestral gene for group 1 *PNPLA*-like genes [*PNPLA1*; *ATGL*; *PNPLA3*/*PNPLA5*; and *PNPLA4*]; *PNPLA6/PNPLA7* for group 2 *PNPLA*-like genes; and *PNPLA8* for group 3 *PNPLA*-like genes (Holmes 2012).

## Summary

The results of this study support previous studies (Wilson et al. 2006; Kienesberger et al. 2009; Saarela et al. 2008; Holmes 2012) for at least eight vertebrate *PNPLA*-like genes and encoded lipases, including five *ATGL*-like genes, namely *PNPLA4* (encoding PNPLA4) and *PNPLA1*, *ATGL* (encoding adipose triglyceride lipase), *PNPLA3* and *PNPLA5* genes; two *PNPLA6*-like genes, *PNPLA6* (encoding neuropathy target esterase) and *PNPLA7*; and *PNPLA8* (encoding cytosolic phospholipase A2). Vertebrate PNPLA4 sequences shared key conserved sequences reported for human PNPLA4 (Gao and Simon 2005; Wilson et al. 2006; Gao et al. 2009), including active site residues, an oxy-anion ‘hole’ sequence, a phosphorylated Thr site and several conserved serine residues. Gene expression data showed that the human *PNPLA4* gene is broadly expressed at higher levels than those for the average gene, for which a CpG island localized in the PNPLA4 promoter and a miRNA binding site localized in the extended 3′noncoding region of *PNPLA4b* mRNA isoform may contribute to these high expression levels. A recent phylogenetic study (Holmes 2012) has suggested that *PNPLA4* is an ancient gene in vertebrate evolution derived from a duplication of an ancestral *ATGL*-like gene within a primitive vertebrate genome.

## Electronic supplementary material

Below is the link to the electronic supplementary material.

Supplementary material 1 (XLS 38 kb)

Supplementary material 2 (XLSX 13 kb)

Supplementary material 3 (XLSX 11 kb)
